# A Segmental 2D Readout Board Manufactured in Printed Circuit Board Technology for Gas Electron Multiplier Detectors

**DOI:** 10.3390/s23198095

**Published:** 2023-09-26

**Authors:** Michał Babij, Piotr Bielówka, Szymon Gburek, Karol Malecha

**Affiliations:** 1 Department of Microsystems, Faculty of Electronics, Photonics and Microsystems, Wrocław University of Science and Technology, wyb. S. Wyspiańskiego 27, 50-370 Wrocław, Poland; 2 Technology Transfer Agency TECHTRA Sp. z o.o., ul. Duńska 13, 54-427 Wrocław, Poland; 3 Centrum Badań Kosmicznych PAN (CBK PAN), ul. Bartycka 18a, 00-716 Warszawa, Poland

**Keywords:** Gas Electron Multiplier (GEM) detector, X-ray, Micro Pattern Gas Detector (MPGD), radiation sensors

## Abstract

The Gas Electron Multiplier (GEM) was introduced by Fabio Sauli in 1997. This technology is broadly used in current and planned High-Energy Physics (HEP) experiments. One of the key components of these detectors is a readout board, which collects charges amplified by GEM foils and transfers them to readout electronics. The commonly used Cartesian XY readout boards are manufactured from the same type of polyamide film used to produce the GEM foils. The manufacturing process utilizes a deep polyimide etching, similar to the Micro Chemical Vias (MCV) etching process, which is protected by patent. The material prepared in this way is glued onto a rigid substrate and mounted in a detector. The production process was developed at CERN, and the technology has been commercialized to a small extent. Consequently, only a few research centers have the ability to make dedicated readout strips readouts. GEM detectors are characterized by a segmented structure that allows the separation of an electron-multiplying structure from a readout. This feature enables the implementation of a new type of charge reading system without the need to interfere with the GEM foil system. A new approach is proposed to simplify production and reduce the costs of GEM detector readout boards. It is based on the concept of segmental readout structures that are manufactured in standard Printed Circuit Board (PCB) technology. The interconnectors and mountings are located on the back of the bottom, so it is possible to place the readout electronics behind the readout plate. The boards are designed in such a way that they can be panelized into a readout with a more extensive active area. The margin between PCBs is minimalized to approximately 200 µm, which is less than 1% of the 70 × 70 mm^2^ board area, so the active area is as big as possible. Therefore, this solution gives us the ability to further increase the size of a readout by adding additional segments, which reduces the cost of scaling up the detector size. A few research groups have suggested similar solutions that utilize PCB technology, but currently, only detectors with 1D zigzag readouts have been validated and used. The measurement results of other 2D (XY) redouts using PCB technology have not been presented. The measurements shown and discussed in this paper validated the proposed technology. X-ray radiographs were obtained, validating the ability to use this technology to manufacture readout boards for GEM detectors. In opposition to state-of-the-art readouts, the proposed solution can be manufactured by any PCB manufacturer without using MCV-patented technology. This gives the users flexibility in designing and ordering low-cost custom readouts.

## 1. Introduction

Micro Pattern Gaseous Detector (MPGD) devices [1] are built from converters of the incident radiation to primary electrons, multiplying structures (Gas Electron Multipliers [2,3,4], micro Resistive Well [5,6,7] and others), readout boards (strip or pixel) and electronic measuring systems. In many cases, multiplying structures and readout boards are fabricated by dedicated technologies like Micro Chemical Vias (MCV), which has been demonstrated in many experiments. However, the patent-protected MCV technique is complicated and requires dedicated competencies and machines. As a result, it is commercialized to a very small extent, and only a few entities are potentially able to produce such sophisticated structures. Consequently, the production costs and time are non-negligible. In many experiments, even advanced experiments such as the CMS GEM tracker upgrade GE1/1 [8], it is necessary to use readout plates with a surface area exceeding 45 m^2^, with a required measurement precision of 200 µm. The average resolution obtained in mass-produced CMS detector modules was measured and found to be 81.3 µm [9]. Even in such advanced installations, the manufacturing of sophisticated circuits made in MCV technology is very expensive and time-consuming. Because of these constraints in many applications, PCB readouts can be considered.

Within the conducted research, a fully operational XY 2D readout board dedicated to GEM detectors with significantly reduced production time and costs was designed, built and tested. A 2D readout with electrodes located on a single plane was proposed as early as 1999 [10], but since that time, only a few designs have been proposed, and even fewer tested. The starting point for this research was an idea presented by A. Zhang. In [11], the author presented the concept of using Printed Circuit Board (PCB) technology to manufacture 2D readout plates. According to this idea, the sensing electrodes are located on a single layer. This design and potential application was also presented in [12]; however, neither prototypes nor test results were presented.

The readout boards manufactured in PCB technology can achieve an active area of at least 2500 cm^2^, but this is not enough for some applications. To overcome this problem, the panelization of several readout boards in a bigger unit was proposed [13]. In the mentioned paper, the authors designed, manufactured and tested the 2D PCB readout and the ability to connect multiple readouts together. However, the obtained resolution was limited to a few mm due to the size of the pads.

In another work, a PCB readout with hexagonal pads in a pitch of 520 µm was proposed [14]. In this case, the resolution was much better than in the previously mentioned solution [13]. Both solutions utilize a UVW readout scheme, instead of XY, in which 50% more ADC channels are needed.

Until now, only one solution to the PCB readout has gained popularity, and this is a ZigZag readout pattern [15]. With this state-of-the-art solution, simple readouts with very dense pitches can be manufactured. The readout works in only one direction (1D), so in 2D imaging, two detectors rotated by 90 degrees are necessary.

The conducted research aimed to optimize and validate the PCB readout geometry using a standard GEM detector. As part of the work, the design assumptions were confirmed, the spatial resolution of the test detector was determined and the possibility of merging a large readout plate from several smaller ones was presented.

Designed PCB readouts were produced within several days by a commercial company. Before the detector assembly, boards were optically inspected and electrically tested. During the checks, no defects were seen, which may indicate that standard PCB shops already have the competencies to produce the proposed structures. Consequently, obtaining more advanced systems directly from industrial partners seems possible. During these tests, the PCB plates were compared with the one made by CERN with MCV technology. The density of the PCB readout strips was about 30% worse than the reference. Nevertheless, the PCB readout boards were fully operational and compatible with GEM detectors. The measurement results are presented in this paper.

Although the measured resolution is slightly lower than that manufactured by MCV technology, it combines the following advantages: the working parameters are sufficient for many applications, the used PCB technology is fast and relatively cheap and it is possible to order readout boards from industrial partners in different sizes and shapes. Finally, it is possible to panelize small boards with a dead area of around 300 µm. The readout active area can be enlarged by merging several independent modules. Therefore, it is possible to adjust the readout size to the actual needs.

This work aimed to develop a new type of Cartesian reading board compatible with GEM detectors manufactured in industrial conditions. A novel XY strip readout dedicated to GEM detectors was designed, built and tested. The proposed solution’s novelty is using standard PCB technology and materials instead of sophisticated and patent-protected MCV techniques. The proposed solution enables the construction of modular, large-area reading boards whose operating parameters do not differ significantly from the most advanced systems. The proposed systems can be produced commercially, significantly reducing production time and costs.

## 2. Materials and Methods

The XY readout board was designed to achieve the best parameters possible (for example, spatial resolution) using standard PCB processes. The design requires laser via drilling technology, which many PCB manufacturers use. The small size of the vias is critical for the readout board pitch size. The surface of this PCB is flat, and it does not contain 3D structures like on a standard XY readout board, so it can be easily used as a substrate for more advanced detectors.

**Figure 1:**
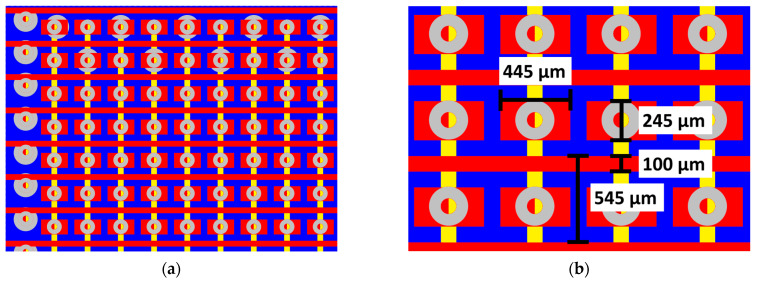
The design of the readout board (the red represents the top layer and the yellow represents the inner layer 1): (**a**) The part showing the *X*-axis lines and the *Y*-axis pads with interconnections between pixels made on inner layer 1; (**b**) the dimensions of the layout features.

The active area of the board is 70 × 70 mm^2^. The design consists of X lines (horizontal) and a series of pads between the X lines. These pads are connected by vertically aligned lines located on a second layer, which forms the Y lines (vertical). The idea of a pixel-line readout structure is shown in Figure 1a. The dimensions of the features are shown in Figure 1b.

The width of the pads is limited by the vias annular ring size, and it measures 245 µm (the hole diameter is 100 µm). With the X trace width and spacing between pads and lines at 100 µm, the minimum achievable pitch is 545 µm. The standard CERN readouts have 390 µm pitch, which is only 28% smaller than the proposed design, so the spatial resolution of the PCB readout should be comparable. The resolution of GEM detectors does not depend linearly on the readout pitch. According to simulations performed for a similar readout, the resolution is 15.6 µm for a 400 µm pitch and between 18.1 µm (500 µm pitch) and 28.9 µm (600 µm pitch) for a 545 µm pitch readout [16]. The experimental data (Table 5, [16]) show a minor difference in resolution—with an increase of pitch from 250 µm to 500 µm, the resolution worsens from 31.5 µm to 46.6 µm. The data shown in [17] show similar values.

The charge cloud is distributed through a few readout lines for readouts with a pitch of 600 µm or less. The algorithm computes the barycenter of the charge cloud and resolves the position. In this case, the further increase in pitch did not linearly result in a resolution increase. Also, it should be mentioned that the spatial resolution is not the most critical parameter for some applications.

**Figure 2:**
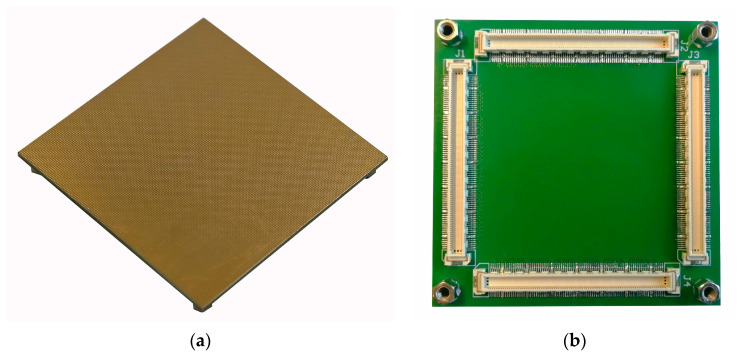
The photos of the 70 × 70 mm^2^ readout board manufactured using PCB technology: (**a**) the top side contains readout electrodes; (**b**) the bottom side contains connectors for the ADC board.

The boards were manufactured by Multi Circuit Boards Limited (Poole, G.B.); 15 pcs. were ordered. This supplier’s turnaround was around 18 working days, which is fast even for the standard readouts manufactured using MCV technology. The board was manufactured using standard FR4 glass-epoxy laminate. It consists of 4 layers, each one made of 35 μm thick copper. The overall PCB thickness was 1.55 mm. The holes between the top layer and the inner layer 1 had a 100 μm diameter and were manufactured using the laser drilling method. Holes on other layers were classically drilled, and their diameter was 150 μm. The blind vias were used on the outer layers, and the buried vias were used between internal layers. The space between traces was only 75 μm. The copper electrodes on the top and bottom sides were coated with chemical gold for passivation. The connectors and mounting poles are located on the bottom side of the board. The standard readout boards utilize 3D structures, so the areas covered by X and Y lines are different to compensate for the charge sharing. There is no need for charge-sharing compensation for electrodes manufactured on a single plane like in the proposed design, so the areas of X and Y electrodes should be the same. Unfortunately, with 245 µm annular rings and a pitch of 545 µm, the Y strips need to be thinner, so the areas of the X and Y electrodes in this design were 54.5 µm^2^ and 109.0 µm^2^, respectively. The photos of the manufactured board are shown in Figure 2. The 70 × 70 mm^2^ dimensions were chosen to achieve 128 × 128 lines with the pitch mentioned above. Also, the size of the connectors limits the readout miniaturization—see Figure 2b. The connectors used were Hirose FX10A-168P-SV (Yokohama, Japan) 168-pin high-density connectors. Bigger readouts (single boards) can be manufactured using this technology if needed.

### 2.1. Panelizing the Readout Boards

**Figure 3:**
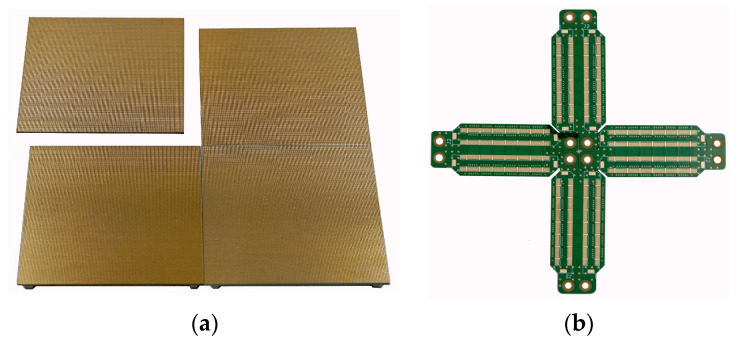
The readout board paneling: (**a**) visualization of a panel assembly using four readout boards; (**b**) interconnection boards used for connecting four readouts in a matrix.

The second design assumption was the ability to connect multiple readout boards together to form more extensive panels. The margin between boards should be as small as possible to reduce the dead area of the readout. Therefore, all the connectors are located on the bottom side of the board, behind the readout electrodes on the top layer. Each signal is routed to two connectors on opposite sides of the board. In this way, one connector can be connected with the readout electronic, and the second connector can be connected to the other readout board using a dedicated interconnection board. The idea of connecting the four readout boards is presented in Figure 3. The dedicated interconnectors used to connect the readout boards are visible in Figure 3b. The *X*-axis lines and the *Y*-axis pads are located around 100 µm from the edges of the readout boards, so with 100 µm of additional clearance between boards, the dead area is smaller than 1%. On the bottom side in the corners, there are 4 M3 mounting posts soldered to the PCB. They should to be screwed to the backplane through the electronic readout or interconnection boards. This ensures the proper alignment between the modules and the strong mechanical connection between detector elements. To prevent stress buildup in the slightly misaligned connectors, the Hirose FX10-168IP-8 (Yokohama, Japan) interposers were used.

### 2.2. The GEM Detector Assembly

**Figure 4:**
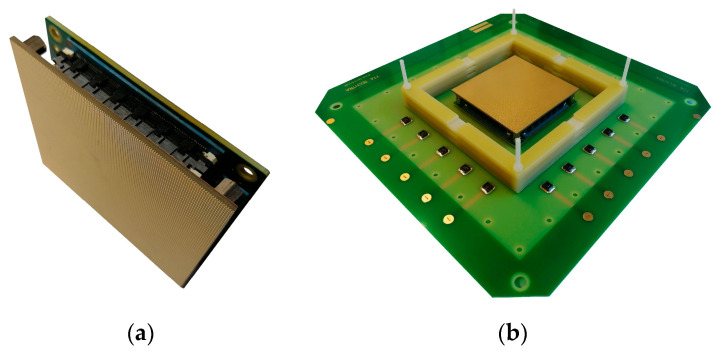
The detector during the assembly: (**a**) a single readout unit consisting of a 256 Ch. electronic ADC board (back) connected with XY readout PCB (front); (**b**) the single readout unit assembled on the GEM detector mainboard.

The dedicated test setup was prepared to validate the readout boards and readout electronics in operational conditions. The complete GEM detector was built using a tested readout, a dedicated backplane and standard CERN 10 × 10 cm detector parts: a set of 3 GEM foils, a drift electrode, spacers for the GEM stack and the gas box. All of these parts were manufactured and assembled by Techtra Sp. z o. o. (Wrocław, Poland). The analogue and ADC parts of the electronic readout coupled with the XY readout board are shown in Figure 4a. The outline of the electronic readout is the same as the readout board, allowing panelization of the detector sectors. The mentioned modules installed on the backplane are presented in Figure 4b. In this image, the frames for the GEM stack and H.V. connectors for the GEM foils can also be seen. The digital part of the readout electronics is located on the outside of the detector gas box and is connected with the ADC module using a 40-pin connector located in the center of a backplane.

## 3. Results and Discussion

The developed readout structures were thoroughly tested to validate the ability to use them in GEM detectors. The boards were visually inspected and electrically tested. One of them was characterized in the functional GEM detector using X-rays. Radiograph imaging was conducted to check the quality of images measured using the designed PCB readout board. The noise of the electronic readout with the readout board was measured. Finally, the charge spread between the X and Y lines was calculated from X-ray measurement data.

### 3.1. Visual Inspection

**Figure 5:**
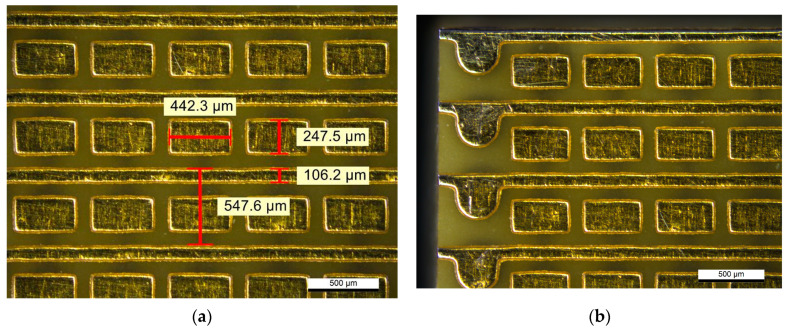
The microscope images of the XY PCB readout board manufactured by one of the PCB manufacturers: (**a**) the line-pad structure with measured dimensions; (**b**) the readout structure near the edges of a board—the distances from structures to the edges are visible.

Ten randomly selected boards were chosen for the visual inspection. The process was carried out using a Leica DM4500 optical microscope (Wetzlar, Germany). The samples were observed in a dark field with 5× magnification to reduce the measurement error. During the inspection, the boards were checked in case of defects: shorts, nonregular pad/trace shape and impurities. The dimensions of the PCB features were also measured. The microscope image of one of the investigated boards is shown in Figure 5a.

The second issue is the distance between the electrodes and the edges of the PCB. This is very important when the individual segments are connected together in a more extensive matrix. Low and consistent distances to the edges lead to achieving a low dead area of the readout matrix. The microscope image of the edges of the investigated board is shown in Figure 5b. All of the investigated boards were of good quality and very consistent. No significant imperfections were spotted. The measured dimensions were very close to the design.

### 3.2. The Electrical Measurements of the PCB Readouts

The electrical measurements were conducted on boards from previous tests. These boards consisted of 128 lines in the X direction and 127 series of pads in the Y direction. All of the boards were tested in case of shorts between the lines. A Keysight U3401A (Santa Rosa, CA, USA) digital multimeter in resistance measurement mode was used to perform these tests. A dedicated adapter board was designed to speed up the measurement process by measuring multiple lines simultaneously. As a result, all of the tested boards were shown to be free of shorts. This confirms that the designed layout can be manufactured accurately and accurately.

**Figure 6:**
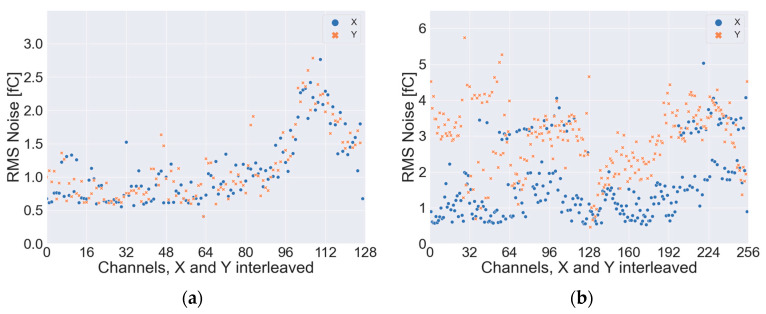
The RMS noise values for each channel of tested readout boards: (**a**) 70 × 70 mm^2^ PCB; (**b**) 100 × 100 mm^2^ CERN.

One randomly chosen board was integrated into a complete GEM detector described in Section 2.2. The noise characteristics of this board were measured using dedicated 128 × 128 channels readout electronics based on DDC2256A charge to digital converter. The noise was calculated using 512 subsequent samples. From these measurements, the mean noise amplitudes, the maximum noise and the RMS values were calculated individually for each channel. The results are shown in Table 1. Measured values were compared to those obtained on the CERN readout board (based on the MCV technology) and Techtra (Wrocław, Poland) 256 × 256 Ch. readout electronics V2.0. The disproportion between noise in the X and Y channels is visible in both cases. This is typical, since the layout of the *X* and *Y* axis is different, so the capacitance of these traces is different. The switching capacitor converters are sensitive to the input capacitance, and the noise floor increases with capacitance. The smaller noise of PCB readout compared to the CERN one can be seen, resulting from the smaller size of the readout board and shorter connections with readout electronics. The RMS noise values for each channel for both tested boards are presented in Figure 6.

### 3.3. Performance Measurements of a GEM Detector with PCB Readout Using X-ray Methods

**Figure 7:**
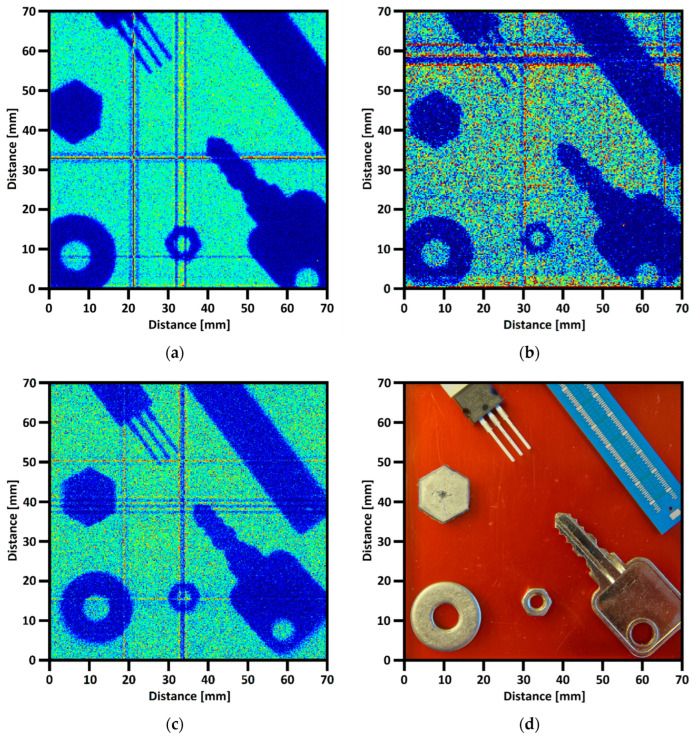
Comparison of the radiographs measured using different readout boards: (**a**) radiograph measured on 70 × 70 mm^2^ PCB board (128 × 127 Ch.); (**b**) radiograph measured on 70 × 70 mm^2^ PCB board (using only 64 × 63 Ch.); (**c**) radiograph measured on CERN 100 × 100 mm^2^ M.S. board [18]; (**d**) objects placed on the detector for the readout board tests [18].

The X-ray measurements of the detector with PCB readout were conducted to validate the imaging quality. The Ar/CO_2_ (70:30) gas mixture was used in these tests as a working gas for the GEMs. This is a standard gas mix used in MPGD. The Ametek COOL-X (Berwyn, PA, USA) pyroelectric X-ray source was used to perform the radiographs. The characteristic photon energies of this source were 8.05 keV (Cu) and 8.14 keV (Ta). The High Voltage that polarizes the stack was set to 3200 V, which gave a detector gain value of 60,000. Several metal and ceramic parts were placed on the detector window during tests. The parts are shown in Figure 7d [18]. The detector with samples was irradiated for about 3 h for each test to obtain enough data. Firstly, the designed PCB readout detector was tested using 128 × 128 channel readout electronics. The obtained radiograph is shown in Figure 7a. In the second experiment, lines were connected in pairs to reduce the readout electronic channel number and to see how they influenced image quality. The resulting image is shown in Figure 7b. As a control image, the measurement was also performed using classic CERN XY readout manufactured using MCV technology (using 256 × 256 channel readout electronics)—see Figure 7c [18].

A few dark ovary bright crossing lines are visible in the images. These image defects are related to the hot pixels. The root cause of hot pixels occurring is the imperfections of the GEM foils or dust particles on the GEM foil surface. The images’ quality and edges’ sharpness are very similar between the measurements. The PCB readout boards seem to be a good alternative to the CERN readout boards, especially in lower-cost applications.

Based on the read data from the detector, the charge-sharing proportion between the X and Y lines was calculated. The charge-sharing compensation in the readout designs is described in Section 2 of this paper. The measurements are based on comparing the charge distribution histograms calculated separately for each axis—this method is described in more detail in [18,19]. The charge histograms were made separately for each axis from 512,000 subsequent samples. Each histogram’s highest peak corresponding to the X-ray source characteristic radiation was located and approximated with Gaussian. The Gauss centers were calculated, and results from individual axes were compared. Table 2 presents the calculated charge distribution data on the developed PCB readout board and the results obtained with CERN readout. The uniformity of the charge in the designed PCB readout was not ideal, but for many applications, the 36.9 to 63.1% ratio is acceptable. Equalization of the charge distribution for the PCB readout requires wider X lines, which implies a higher pitch, or requires the reduction of the hole sizes and pads of the *Y* axis, which could be achieved, but not by any standard PCB manufacturers.

## 4. Conclusions

The simplified 2D readouts manufactured using standard PCB technology seem to be a suitable solution for the GEM detectors, especially for low-cost solutions or in applications where readout boards without margins are beneficial. A 70 × 70 mm^2^ PCB was designed and manufactured for this test, but much bigger readouts can be easily mass-manufactured using PCB technology. The prototype boards were thoroughly tested using optical and electronic measurements. One of the mentioned readouts was assembled into a test detector and validated using X-rays. The obtained results were compared to ones measured using standard CERN boards manufactured using MCV technology. The noise performance of those XY readouts was very good—the noise level was low, and it was very uniform. The imaging ability of the detector with this board was confirmed, and the obtained spatial resolution and quality of the image were comparable to state-of-the-art solutions. The active area covered almost all of the board, so connecting the single readouts into a bigger matrix was possible with less than 1% dead area. The charge distribution between the *X* and *Y* axis was in the proportions of 36.9 to 63.1%, which is acceptable for most applications. It could be equalized using more advanced technology or using designs with bigger pitches.

This work shows that the parameters of the PCB readouts are similar to those achieved using MCV technology, and can be used alternatively in most applications. Utilization of simpler and commercially available methods will reduce costs and lead time and give the user more flexibility in designing custom readout boards. This method also allows easy integration of readout boards with electronic readout on the same PCB, which can reduce the trace lengths and noise level.

## Figures and Tables

**Table 1:** Noise characteristics of the detector with PCB readout board compared to CERN readout board manufactured with MCV technology.

Board	Channels	Avg. Noise [fC]	Max Noise [fC]	RMS Noise [fC_RMS_]
PCB 70 × 70 mm^2^	X (TOP)	2.38	4.10	1.10
	Y (BOT)	2.47	6.15	1.14
CERN 100 × 100 mm^2^	X (TOP)	4.04	22.27	1.63
	Y (BOT)	5.24	14.25	2.89

**Table 2:** Charge distribution between the top and bottom electrodes of the PCB readout board compared to the CERN readout board.

Board	Channels	Gauss Center [fC]	Total Charge [%]
PCB 70 × 70 mm^2^	X (TOP)	4.12	36.9
	Y (BOT)	7.05	63.1
CERN 100 × 100 mm^2^	X (TOP)	1.48	56.3
	Y (BOT)	1.15	43.7

## Data Availability

Not applicable.
